# Ultrasonic biomicroscope-guided radiofrequency ablation of the ciliary body in a rabbit glaucoma model

**DOI:** 10.1038/s41598-025-23784-4

**Published:** 2025-11-14

**Authors:** Jinglan Li, Baoke Hou

**Affiliations:** 1 Senior Department of Ophthalmology, The Third Medical Center of PLA General Hospital, Beijing, China; 2 Department of Ophthalmology, The First Medical Center of PLA General Hospital, Beijing, China; 3 Department of Ophthalmology, General Hospital of Southern Theater Command of PLA, Guangzhou, China

**Keywords:** Radiofrequency ablation, Ciliary body, Glaucoma, Histopathology, Diseases, Medical research, Pathogenesis

## Abstract

Destruction of the ciliary body is regarded as a potential way to lower intraocular pressure (IOP) and treat glaucoma patients. This study evaluated the IOP-lowering capability of a novel device for ciliary body radiofrequency ablation (RFA) in a rabbit glaucoma model. The mechanism of RFA-induced IOP reduction was also investigated by evaluating its histological effects. Thirty rabbits were equally divided into the model (*n* = 10: silicone oil injected eyes), sham (*n* = 10: silicone oil injected eyes in which sham RFA was performed), and RFA (*n* = 10: silicone oil injected eyes in which RFA was performed) groups. The glaucoma model was established by injecting silicone oil into the anterior chamber of the rabbit’s eye. The temperature of the ablation point in the ciliary body during RFA was recorded using an infrared thermal imager, and the changes in IOP after surgery were compared among groups. The rabbits were monitored for postoperative complications, and histological examinations were performed at 1 week, 1 month, and 6 months after surgery. The success rate of the rabbit eye glaucoma model was 86.7%. Modeling induced an approximately 2.24-fold increase in IOP (from 13.67 ± 1.68 to 30.73 ± 7.68 mmHg) within 1 week. Compared with that in the sham group, the IOP in the RFA group decreased significantly at all follow-up time points (*P* < 0.01). The overall IOP amplitude decreased by approximately 50%, with the final IOP maintained at approximately 11 mmHg. During RFA, the ablation point temperature increased to 62.53 ± 5.05 ℃. Ultrasonic biomicroscopy after RFA showed ciliary body tissue defects at the ablation site. The ciliary processes were reduced in number, sparsely arranged, and irregularly shaped. Hematoxylin and eosin staining revealed coagulative necrosis of the ciliary processes in the ablation zone, and the bilaminar cells on the ciliary process surface were replaced by dimorphic and dysfunctional non-bilaminar epithelium. Scanning electron microscopy and transmission electron microscopy showed loss of epithelial cells, and the cytoplasm contained fewer mitochondria, which were swollen. The nucleus and chromatin were condensed, indicating cell death and apoptosis. A TdT-mediated dUTP nick-end labeling assay confirmed apoptosis of some epithelial cells. Ciliary body RFA appears to be a feasible, effective, and well-tolerated method of lowering IOP in the rabbit glaucoma model.

## Introduction

Glaucoma is the leading cause of irreversible blindness worldwide. A traditional approach to treating refractory glaucoma is destruction of the ciliary body to reduce aqueous humor production, which decreases intraocular pressure (IOP), relieves ocular pain, and preserves residual visual function^1^. Attempts to reduce aqueous humor synthesis employing ciliary body destruction are by no means novel. Different methods and energy sources for ciliary body destruction have been studied and applied^2,3^, all resulting in coagulative necrosis of the ciliary body following cyclocryotherapy or ciliary body heating. However, these methods have various limitations that restrict their clinical application^1–3^. First, they generally lack selectivity for the target organ, often damaging adjacent tissues (e.g., iris, retina), causing ocular inflammation, and even resulting in vision loss. Previous research has reported that the risks of vision loss, intraocular inflammation, chronic inflammation, corneal dystrophy, cataract, and surgical failure range from 6 to 64.3%, 0.5 to 37.5%, 12.4 to 27%, 2 to 6%, 10 to 35%, and 12.9 to 80%, respectively, at 1 year after surgery^4–12^. Second, the dose–response relationship of these methods is unpredictable, potentially leading to uncontrolled IOP after ciliary body destruction. Additionally, for clinical application, treatment methods need to be simple to operate and cost effective. Therefore, there is an urgent need to develop more safe, effective, simple, and affordable therapeutic approaches for glaucoma.

Radiofrequency ablation (RFA) technology, initially described by D’Arsonval in 1891, uses radiofrequency waves to elevate the tissue temperature^13^. By altering the radiofrequency current, high-frequency vibrations induced by the electrode cause ion agitation and friction within the surrounding tissue, generating frictional heating^14^. This thermal effect endows the radiofrequency treatment instrument with ablative and cutting functions. Compared with traditional surgical treatments, RFA has advantages such as a shorter treatment time, less bleeding, less invasiveness, and more precise control^15^. RFA is widely recognized by physicians in clinical practice, particularly ultrasound-guided percutaneous RFA, which has been extensively used to treat arrhythmias, herniated discs, gynecological diseases, and various tumors (such as osteoid osteoma, hepatocellular carcinoma, renal cell carcinoma, hyperfunctioning parathyroid adenoma, and tumor metastasis)^16–20^. Currently, RFA is also used in the field of ophthalmology to treat presbyopia, keratoconus, orbital tumors, and trichiasis^21–25^, indicating its safety and efficacy as a treatment for ocular diseases. However, RFA has not been used to treat glaucoma.

**Fig. 1:**
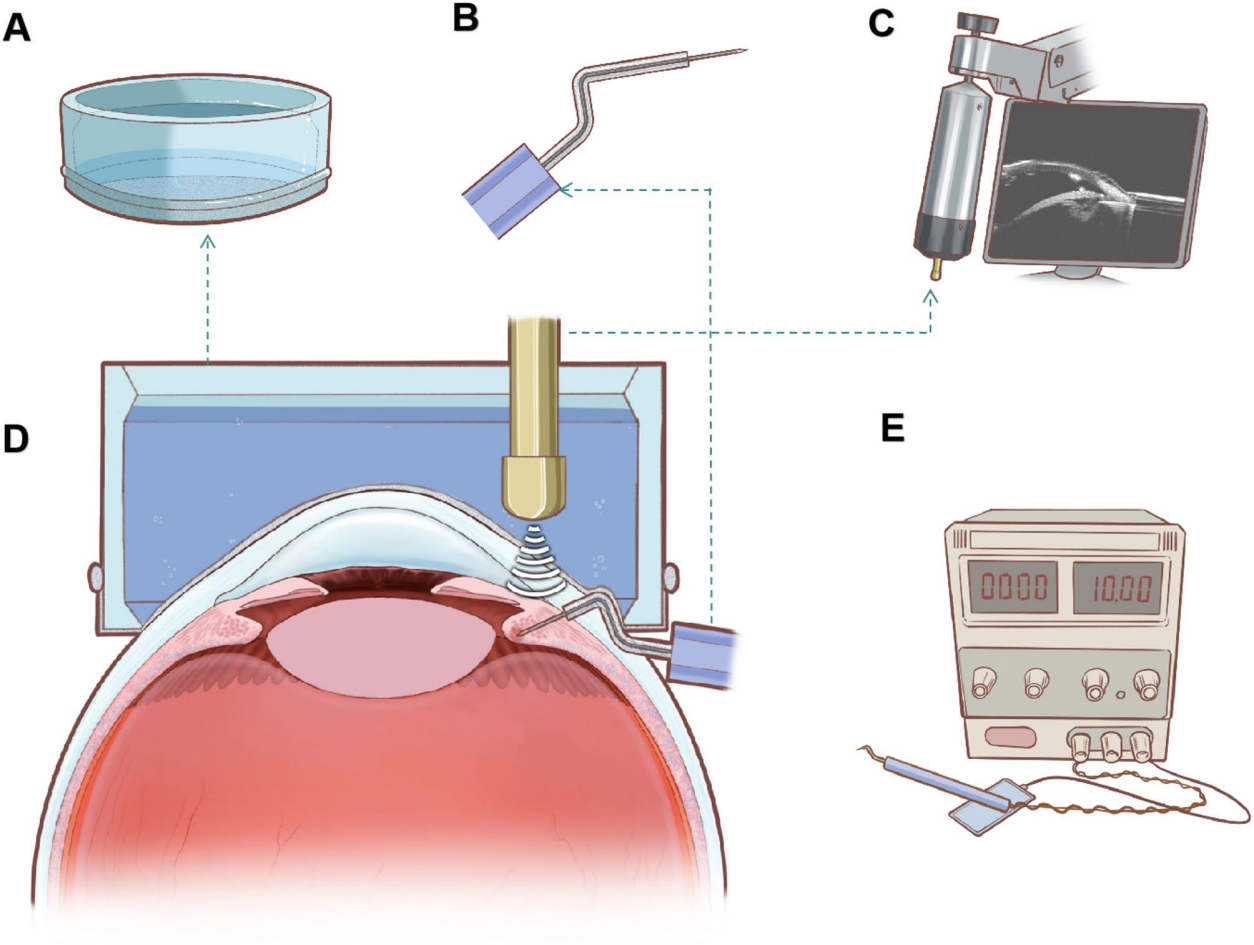
An illustration of the ciliary radiofrequency ablation (RFA) device. (**A**) The self-designed lens-water bath transformation system (Patent No.: ZL201420207350.0), whose bottom is made of a special material with waterproof and flexible properties. (**B**) The puncture electrode is sharp, and the rear section is wrapped with the insulating sheath, so that the ablation effect focuses around the tip of the puncture needle, avoiding ablation damage to the puncture path. (**C**) Ultrasonic biomicroscope guides real-time observation of RFA needle placement and ablation extent. (**D**) Ciliary radiofrequency ablation cross-section diagram. (**E**) The voltage regulator, ablation energy output device and display panel are combined into an integrated machine, which can adjust the ablation frequency and radiofrequency output mode.

Inspired by these theoretical foundations and clinical experiences, our team designed an ultrasonic biomicroscope (UBM)-guided ciliary body RFA device (Fig. 1) for refractory glaucoma treatment. Employing self-designed RFA electrodes, this minimally invasive interventional therapy uses UBM guidance for precise ciliary body tissue ablation, allowing the number of ablated ciliary processes to be controlled. Our previous study found that RFA was safe and effective for reducing IOP in normal rabbit eyes^26^. However, the long-term histological effects of RFA in a glaucoma model and the details of the mechanism by which RFA lowers IOP have not been elucidated.

Therefore, in this study, a rabbit glaucoma model was established based on existing literature^27–30^. IOP reduction trends, histological effects, and ocular complications after UBM-guided RFA were analyzed. In particular, the macroscopic and microscopic manifestations of the ciliary body lesions at different stages, as well as the adjacent tissue damage, were evaluated.

## Result

### Injection of silicone oil into the anterior chamber increased IOP

**Fig. 2:**
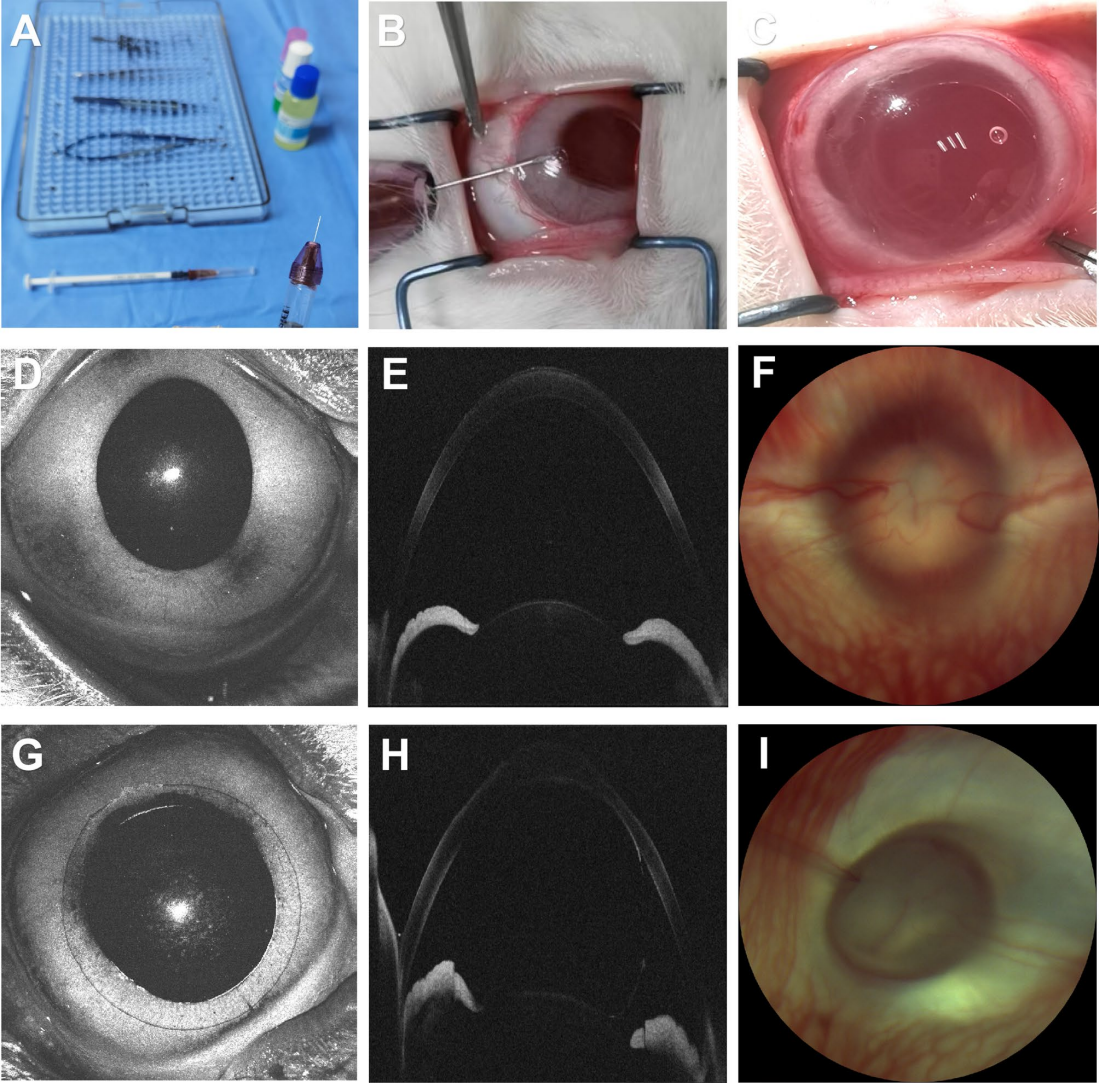
Establishment and validation of rabbit glaucoma model. (**A**) The instruments used for molding. (**B**) Inject silicone oil into the anterior chamber. (**C**) After modeling, the oil droplet expanded to cover about 80% of the anterior chamber. (**D**, **G**) Photos of the rabbit anterior segment before and 1 week after molding. (**E**, **H**) Anterior segment OCT imaging before and 1 week after molding. Under the pressure of silicone oil, the root of the iris contacts the posterior surface of the cornea, and the anterior chamber angle closes. (**F**, **I**) Fundus photography before and 1 week after molding.

Approximately 150 μL of silicone oil was injected into the anterior chamber of the right eye of each rabbit, filling about 80% of the anterior chamber and completely covering the pupil. After 2–3 days, the silicone oil coalesced into spherical droplets due to its surface tension, and pupil dilation was observed (Fig. 2C). As illustrated in Fig. 2, the spherical droplets contacted the iris surface, blocking the pupil. The ciliary body continuously produces aqueous humor, which accumulates in the posterior chamber and pushes the iris forward. When the root of the iris contacts the posterior surface of the cornea, the anterior chamber angle closes, which was confirmed by anterior segment optical coherence tomography (AS-OCT) (Fig. 2H). Angle closure further hinders aqueous humor outflow through the trabecular meshwork and can also contribute to IOP elevation.

A characteristic morphological feature of human glaucoma is the enlargement of the cup-disc ratio, and also cupping of the optic disc. This characteristic change was detected in the model using fundus photography and was accompanied by pale ischemia of the optic disc, corresponding to increased IOP (Fig. 2I). These results indicated that intracameral silicone oil injection can induce an effective pupillary block in rabbits and mimic the characteristics of acute angle-closure glaucoma.

### IOP and ophthalmic examinations

One week after glaucoma modeling, the IOP increased by a factor of approximately 2.24 (from 13.67 ± 1.68 mmHg to 30.73 ± 7.68 mmHg, *P* < 0.01), and the modeling success rate was 86.7%. The high IOP was maintained at about 30 mmHg for more than 1 month. Compared with that in the model group, the IOP in the sham group did not change significantly (30.73 ± 7.68 mmHg *vs.* 31.95 ± 6.87 mmHg, *P* = 0.90), indicating that the minor damage and stimulation to the eye caused by the electrode puncture did not cause significant changes in IOP. However, compared with that in the sham group, the IOP in the RFA group decreased significantly at all follow-up time points (*P* < 0.01). Compared with the IOP of the RFA group at 1 week after modeling, although there was no significant difference in IOP at 3 days or at 1 week after RFA (*P* = 0.41 and *P* = 0.22, respectively), a significant reduction in IOP was observed at 2, 3, 4, and 8 weeks after RFA (*P* < 0.01). The overall amplitude of the IOP decreased by about 50%. The final IOP after RFA was maintained at approximately 11 mmHg. (Table 1).

**Table 1:** IOP in the glaucoma model, sham and RFA groups during the follow-up.

	Model group	Sham group	RFA group	*P* (Model vs. Sham)	*P* (RFA vs. Sham)	*P* (vs. 1 week after modeling)
Before modeling	13.67 ± 1.68	13.64 ± 2.06	13.37 ± 2.24	0.99	0.10	< 0.01
1 week after modeling	30.73 ± 7.68	31.95 ± 6.87	28.63 ± 8.30	0.90	0.47	–
3 days after treatment	29.74 ± 7.39	29.17 ± 7.39	18.86 ± 5.16	0.99	< 0.01	0.41
1 week after treatment	29.69 ± 7.74	30.57 ± 7.80	20.47 ± 8.60	0.95	< 0.01	0.22
2 weeks after treatment	29.41 ± 8.11	29.71 ± 7.45	13.56 ± 5.74	0.99	< 0.01	< 0.01
3 weeks after treatment	31.19 ± 8.02	29.03 ± 6.91	11.66 ± 4.39	0.99	< 0.01	< 0.01
4 weeks after treatment	28.89 ± 6.89	28.35 ± 6.38	11.69 ± 4.12	0.99	< 0.01	< 0.01
8 weeks after treatment	–	–	11.73 ± 4.06	–	–	< 0.01

No visible abnormalities were observed macroscopically during any of the surgeries. No endophthalmitis or phthisis occurred during follow-up. No rabbits died during the study. Until 1 month after treatment, the anterior segment and retina showed no abnormalities, and the optic disc presented glaucomatous alterations of different degrees. However, 1 month after treatment, the corneas of model group and sham group exhibited damage due to prolonged exposure to elevated IOP. Gradual corneal thickening and opacification were observed, which can compromise the accuracy of IOP measurements. So these IOP data were not included in the statistics.

### UBM investigation during and after RFA

**Fig. 3:**
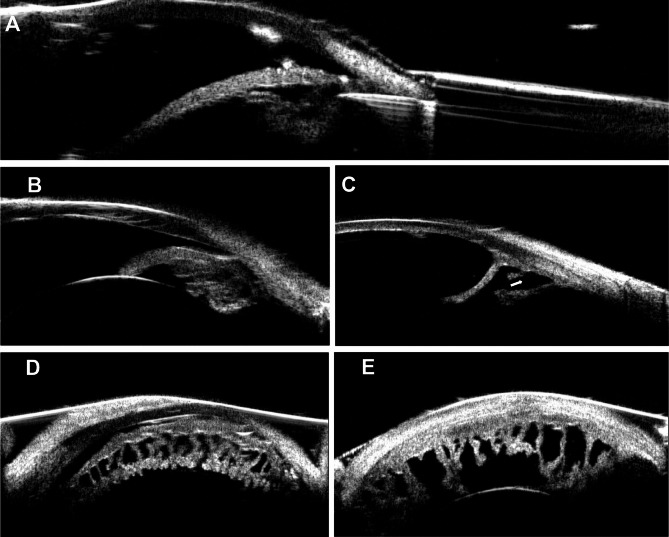
Ultrasonic biomicroscope (UBM) investigation during and after RFA. (**A**) A real-time RFA image of UBM showed that the electrode was positioned in the ciliary process through the sclera without contacting the anterior chamber angle and the iris. (**B**) The radial scanning of UBM showed that iris root was attached to the cornea and the anterior chamber angle was closed in silicone oil-induced intraocular hypertension model. (**C**) The ciliary body tissue was defective at the ablation site with radial scanning of UBM, and the white arrow points to the ciliary defect. (**D**) Horizontal scanning of ciliary before RFA. (**E**) Horizontal scanning showed that the ciliary bodies were reduced in number, sparsely arranged, and irregularly shaped after RFA.

The relationship between the puncture electrode and the ciliary body was monitored in real time by using a lens–water bath transformation system combined with UBM to ensure the appropriate position and depth of ablation. This method was used to observe changes in the anterior segment at different time points after ablation, including changes in the cornea, chamber angle, iris, and morphology and quantity of ciliary processes. Figure 3A showed a real-time image of RFA in which the electrode was positioned in the ciliary body through the sclera without contacting the anterior chamber angle or the iris. In addition, the iris root was attached to the cornea, and the anterior chamber angle was closed in the silicone oil–induced intraocular hypertension model (Fig. 3B). Compared with the contralateral non-ablated ciliary body (Fig. 3B, D) 1 month after RFA, the ciliary body volume was significantly reduced, and ciliary body tissue defects were observed at the ablation site (Fig. 3C) according to radial scanning. Horizontal scanning showed that the ciliary processes were reduced in number, sparsely arranged, and irregularly shaped (Fig. 3E).

### Tissue temperature monitored during RFA

**Fig. 4:**
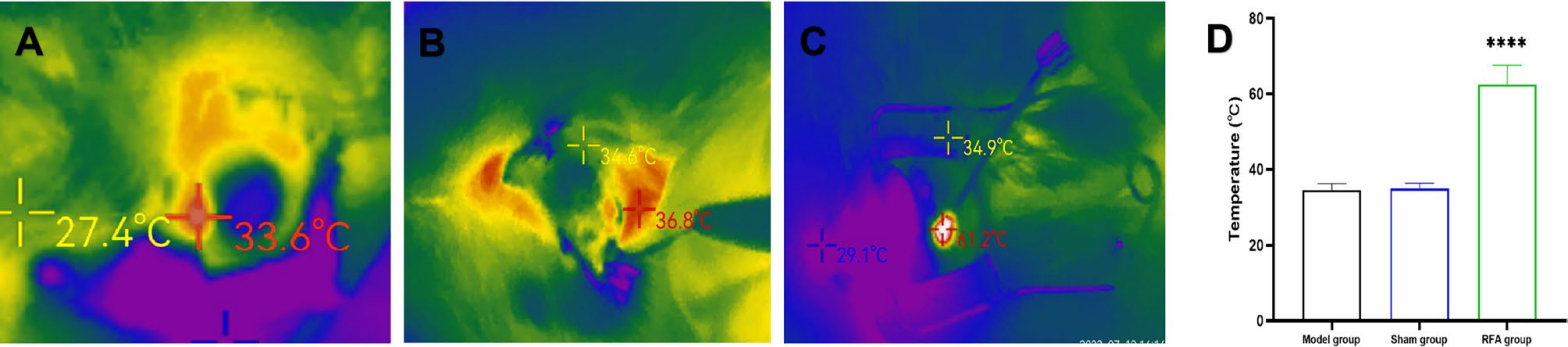
Infrared thermography detects temperature changes during RFA. (**A**) Infrared temperature measurement images of the model group, the local maximum temperature during this ablation was recorded as 33.6℃. (**B**) Infrared temperature measurement images of the sham group, the local maximum temperature during this ablation was recorded as 36.8℃. (**C**) Infrared temperature measurement images of the RFA group, the local maximum temperature during this ablation was recorded as 61.2℃. (**D**) Statistical analysis of RFA temperatures for each group. ****P < 0.0001.

The changes in ablation point temperature during RFA were observed by real-time video recording with an infrared thermal imager, and the local maximum temperature during each ablation process was recorded and analyzed. Statistical analysis (Fig. 4D) showed that the temperature was 34.64 ± 1.64 °C in the model group (Fig. 4A), 34.97 ± 1.44 °C in the sham group (Fig. 4B), and 62.53 ± 5.05 °C in the RFA group (Fig. 4C). The rate of temperature increase was consistent with the duration of ablation. The local temperature was highest at the end of a single RFA session and rapidly decreased to normal body temperature within a few seconds after the RFA.

### Haematoxylin and eosin staining (HE) and histopathological changes

**Fig. 5:**
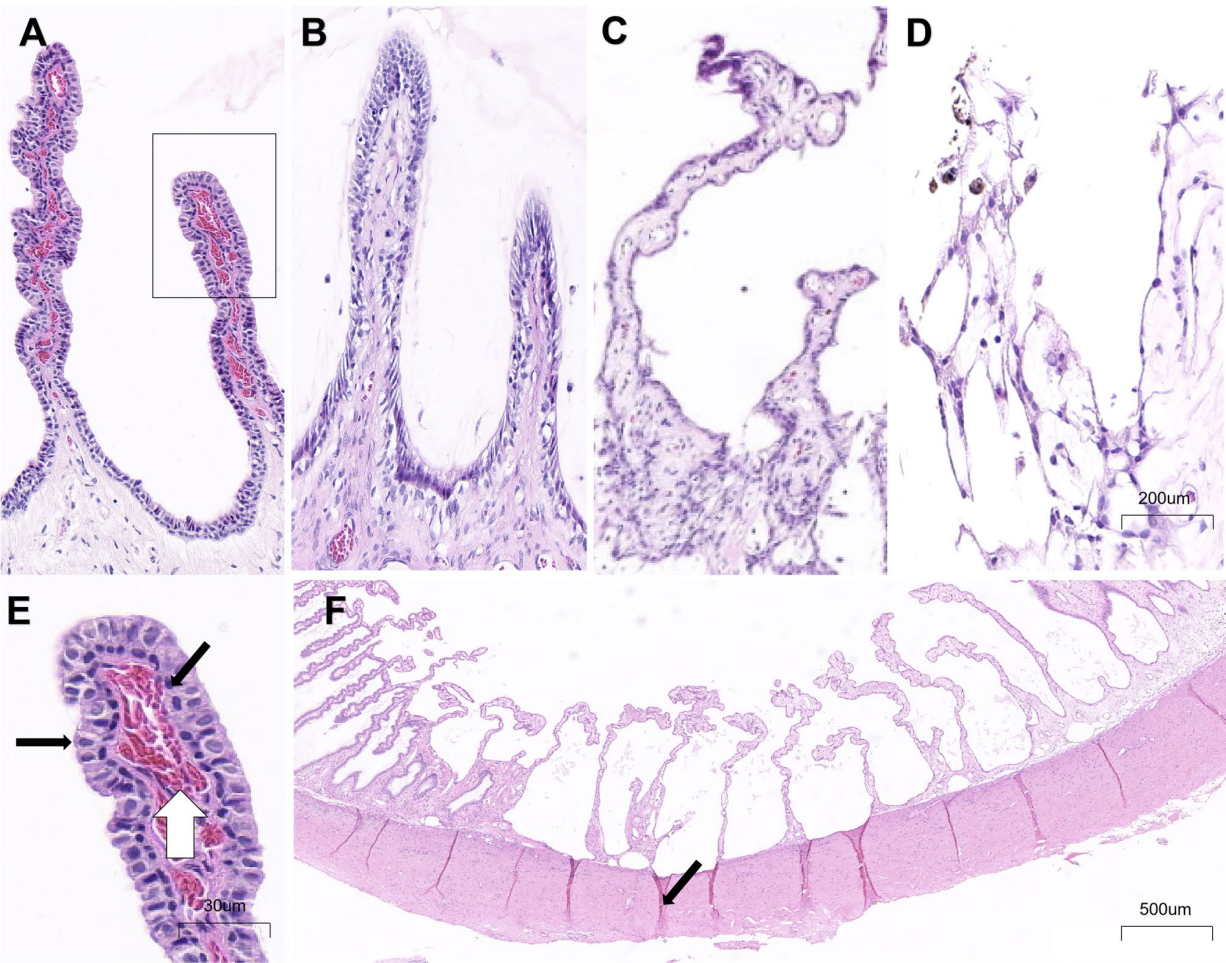
HE staining after RFA. (**A**) The normal ciliary processes. (**B**) HE image of the ciliary processes at 1 week after RFA. (**C**) HE image of the ciliary processes at 1 month after RFA. (**D**) HE image of the ciliary processes at 6 months after RFA. (**E**) Enlarged image of the normal ciliary process. The black horizontal arrow points to non-pigmented epithelial cell, the black oblique arrow points to pigmented epithelial cell, and the white arrow points to the ciliary stroma and blood vessel. (**F**) HE image of some adjacent ciliary processes at 1 month after RFA. The arrow indicates the thinning of the sclera.

Histologic examination of the treated eyes at 1 week, 1 month, and 6 months showed coagulative necrosis of the ciliary processes in the ablation zone (Fig. 5), which was most pronounced in the distal and middle ciliary processes. In the RFA areas, the ciliary processes showed inflammatory cell infiltration, stromal edema (marked dilation of collagen fibers), vascular occlusion, epithelial cell coagulation necrosis with cell loss, and hemorrhage. The inflammatory cell reaction was mild, with a small number of macrophages, lymphocytes, plasma cells, polymorphonuclear cells, and giant cells present. The area width of the three RFA sites was about 4 ± 0.5 mm (about 10 to 16 adjacent ciliary processes) (Fig. 5F).

From months 1–6, HE histological examination showed progressive degeneration and atrophy of ciliary processes and expansion of interstitial collagen fibers. The bilaminar cells on the ciliary process surfaces were replaced by dimorphic and nonfunctional non-bilaminar epithelium (Fig. 5C, D). We also observed a thinning of the sclera near the electrode puncture point in the RFA area (Fig. 5F), and the thinnest sclera thickness was approximately 69 ± 3% of the unaffected sclera thickness (305 ± 26 µm *vs.* 441 ± 21 µm).

In the RFA group, there was one case (1/10) of focal choroidal hemorrhage and subretinal hemorrhage, which was probably because the location or direction of the electrode puncture was closer to the retina. The other rabbits showed no signs of retinal necrosis, retinal detachment, choroidal detachment, or choroidal hemorrhage.

### Scanning electron microscopy (SEM) analysis

**Fig. 6:**
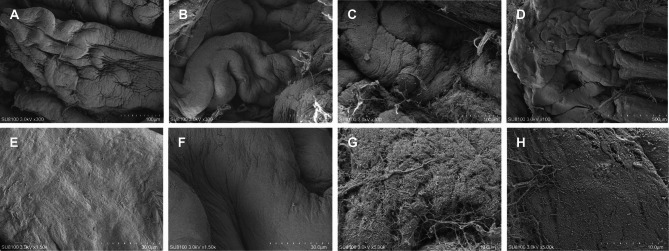
Scanning electron microscopy images of ciliary process. (**A**, **E**) The normal ciliary process. (**B**, **F**) The ciliary process of model group. (**C**, **D**, **G**, **H**) The ciliary process of RFA group.

SEM showed that ciliary processes without ablation were full in shape (Fig. 6A). Due to the high IOP, the ciliary processes in the sham group were slightly distorted (Fig. 6B), but their surfaces were as smooth as those of normal ciliary processes (Fig. 6E, F) and covered by regular grooves and folds composed of epithelial cells (Fig. 6B). One week after RFA, the ciliary processes were mutated and thickened due to stromal edema, and the grooves and folds on their surfaces decreased in number and became shallower (Fig. 6C). High magnification images showed loss of the bilayer epithelium on the basement membrane, exposure of the rough basement membrane, disorder of tissue fibers, and a disorganized epithelium composed of dysmorphic and flattened cells covering the surface (Fig. 6G, H). The ciliary processes degenerated and atrophied (Fig. 6D).

### Transmission electron microscopy (TEM) analysis

**Fig. 7:**
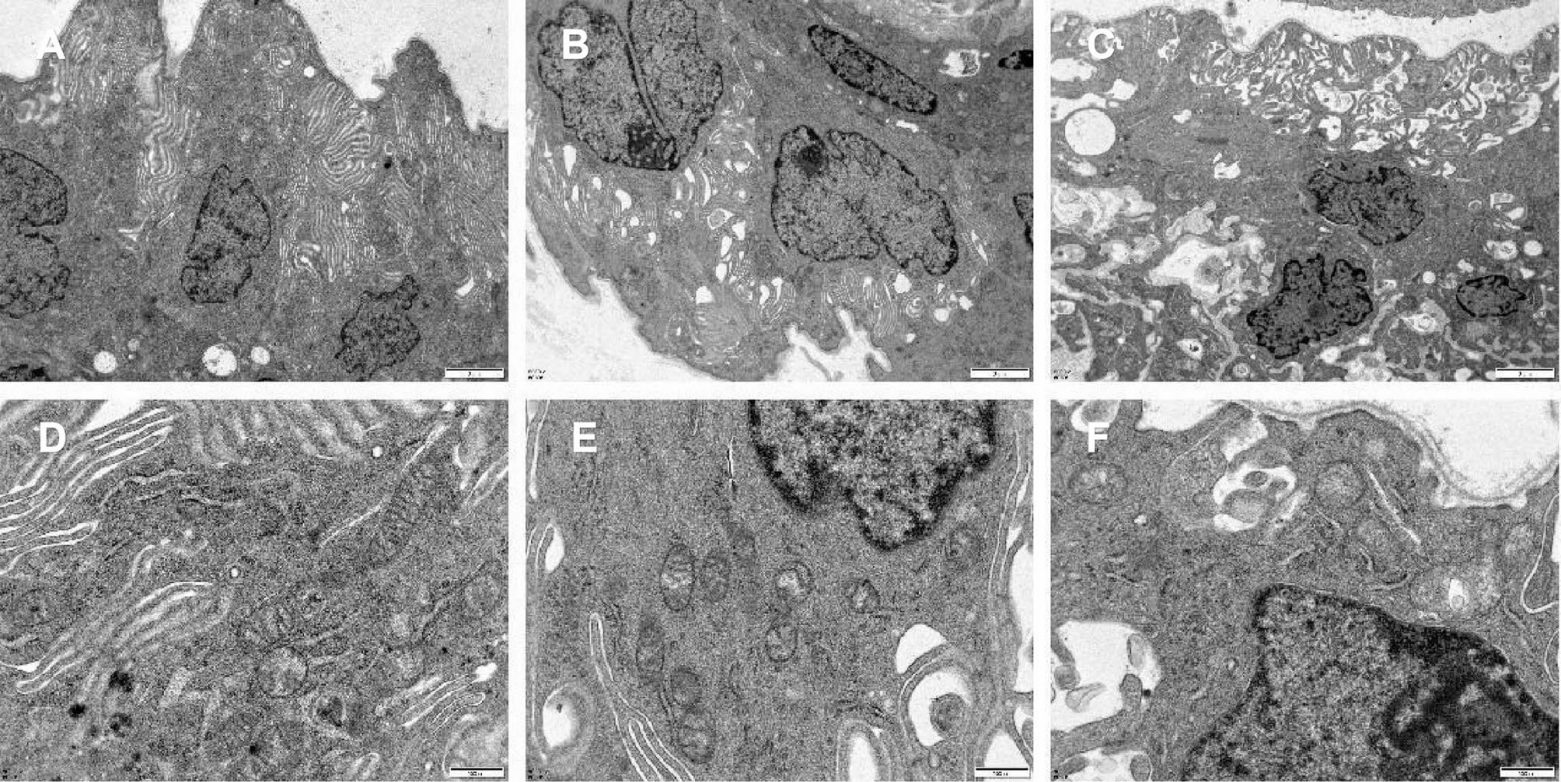
Transmission electron microscopy images of ciliary process. (**A**, **D**) The normal ciliary process. (**B**, **E**) The ciliary process of model group. (**C**, **F**) The ciliary process of RFA group.

TEM at 1 week after RFA showed that the ciliary body epithelium after RFA appeared thinner, with fewer apical cilia and more vacuolar changes (Fig. 7C), than the non-ablated ciliary body epithelium (Fig. 7A, B). The cytoplasm of the non-pigmented cells contained fewer mitochondria, and these mitochondria were swollen and irregular in shape (Fig. 7F). In contrast, the cytoplasm of the non-pigmented cells in the sham group (Fig. 7E) and the normal control group (Fig. 7D) contained more mitochondria, with normal morphology. Moreover, the ciliary process epithelial cells showed nuclear pyknosis, chromatin condensation, necrosis, and apoptosis after RFA (Fig. 7C, F).

### TdT-mediated dUTP nick-end labeling (TUNEL) staining analysis

**Fig. 8:**
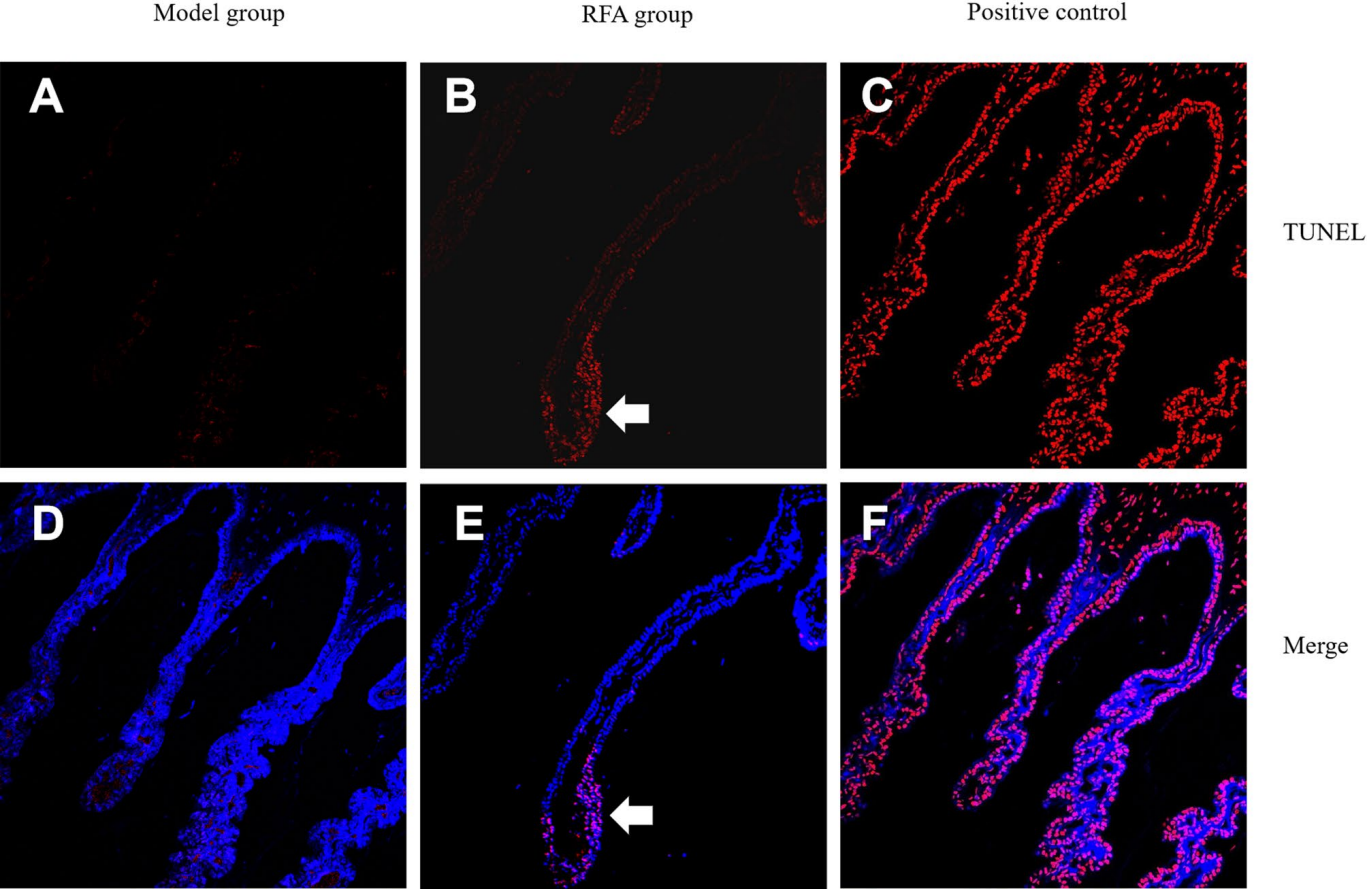
TUNEL staining images of ciliary process. (**A**, **D**) The ciliary process of model group. (**B**, **E**) The ciliary process of RFA group. The arrow indicates that the nucleus of the cell in the apoptotic state turns red under TUNEL staining. (**C**, **F**) The positive control of ciliary process.

One week after RFA treatment, some of the ciliary processes completely disappeared, and the surrounding ciliary processes that were not directly ablated were edematous and thickened, especially the distal and middle ones. The bilateral epithelial cell structure still existed, but the nuclei were positive for TUNEL staining, indicating that the cells were damaged and in a state of apoptosis (Fig. 8).

## Discussion

This study aimed to evaluate the extent and mechanism of IOP reduction after ciliary body RFA in a rabbit model of glaucoma by analyzing the histological changes in the ciliary body and the damage to adjacent tissues. We evaluated the potential of a novel RFA device to reduce IOP in a rabbit glaucoma model via ciliary body destruction. The design of the puncture electrode, which was based on a previous study^26^, was optimized by sharpening the puncture needle and adding an insulating sheath to protect the sclera tissue more effectively (Patent No.: CN201420058061) and concentrate the ablation energy on the ciliary processes. RFA was combined with real-time UBM monitoring using a lens–water bath transformation system. This approach controls the reduction in IOP through the localization and quantitative ablation of the ciliary body under UBM guidance.

Currently, the most commonly used methods of ciliary body destruction in clinical practice include transscleral cyclophotocoagulation (TCP), endoscopic cyclophotocoagulation (ECP), and high-intensity focused ultrasound (HIFU) cyclocoagulation. Lasers serve as the energy source in TCP and ECP, and their energy concentration and tissue heating depend on tissue pigmentation. This may result in uncontrolled energy absorption and potentially affect the richly pigmented tissues surrounding the ciliary processes, such as the iris and trabecular meshwork, leading to anterior chamber inflammatory reactions and unpredictable IOP after treatment. ECP uses direct visualization to improve the targeting of the ciliary processes, but the inherent disadvantages of lasers and the risks of intraocular surgery have limited its clinical application. In recent years, many studies have been conducted on HIFU cyclocoagulation^31–36^, which is not affected by pigmentation and can heat tissue at a specific location and depth, improving the targeting and controllability of ciliary body destruction. However, the complexity of equipment operation and high economic cost have hindered its widespread application. Compared with these methods of ciliary body destruction, the novel device has several advantages: UBM is used to locate the ciliary processes, preventing damage to nearby tissues; the energy deposition and tissue heating at the RFA focus site are not affected by tissue pigmentation, preventing other pigmented tissues in the eye from absorbing energy; and the RFA energy can be adjusted to heat and treat a defined and controlled tissue area.

The damage in the treatment area was highly concentrated in the location area, which may account for the absence of observed damage to adjacent ocular tissues. In one rabbit of the RFA group, we observed focal choroidal hemorrhage and subretinal hemorrhage. During the RFA treatment of this rabbit, we had difficulty in anesthetizing this rabbit, which resulted in the rabbit’s moving and contracting the eyelids. These movements might have led to the misplacement of the electrode puncture point, resulting in richly vascularized choroidal and retinal hemorrhage after RFA. In other rabbits, no signs of retinal necrosis, retinal detachment, choroidal detachment or hemorrhage were observed.

Human cells can survive at a temperature of approximately 42 °C. At 42 °C–45 °C, cells suffer damage after exposure for 30 to 60 min^37^. RFA can increase the local tissue temperature to over 50 °C, causing coagulative necrosis via thermal ablation. In this case, a short period (a few seconds or minutes) is enough to cause irreversible thermal damage to the cells. At temperatures above 100 °C, the main water content of the target tissue undergoes vaporization, and as the temperature rises, subsequent carbonization and smoke generation occur^20,38,39^. The highest temperature in the ablation area was 62.53 °C ± 5.05 °C, which was sufficient to cause coagulative necrosis of the local tissue. During RFA, the tissue adjacent to the puncture electrode tip (the central region) experienced burn erosion–induced damage, and the surrounding ciliary processes also suffered corresponding thermal damage due to heat conduction from the central region. Therefore, tissue damage is not limited to the puncture site but also includes ciliary processes in the surrounding region.

One week after RFA, we observed coagulative necrosis of the ciliary processes in the ablation zone, while there was no obvious damage in the non-ablated area. Six months later, the bilaminar cells on the ciliary process surfaces were replaced by dimorphic and dysfunctional non-bilaminar epithelium, and the IOP was significantly lowered and stably maintained. Therefore, we hypothesized that IOP reduction after RFA may occur because the damage to the bilaminar epithelium on the ciliary processes reduces aqueous humor generation. New Zealand rabbits typically have 100 to 120 ciliary processes, of which approximately 50–70 were destroyed by RFA in the experiment, while the uninjured ciliary processes could still maintain aqueous humor secretion. SEM and TEM, used to observe the microscopic features of the ciliary processes in the ablation zone, showed a loss of epithelial cells and a decrease in the number of cilia. The cytoplasm contained fewer mitochondria, many of which exhibited signs of swelling. The nucleus and chromatin were condensed, indicating apoptosis; TUNEL staining confirmed that some epithelial cells had undergone apoptosis.

Many mechanisms have been hypothesized to explain the reduction in IOP after ciliary body damage. For instance, damage to pigmented and non-pigmented epithelium reduces aqueous humor generation, causes ciliary body inflammation, enhances outflow through thinned sclera, and increases uveoscleral outflow due to damage in the ciliary body stroma and pars plana^40–42^. Similar findings have been confirmed in both rabbits and humans^36,43,44^. Our histological and ophthalmological examination of rabbits also revealed damage to the ciliary body epithelial cells and thinning of the sclera, but did not show obvious inflammation. Different techniques for destroying ciliary processes may have different mechanisms. These observations can inform further optimization of the device to increase the safety and stability of IOP reduction.

This study had several limitations. First, one concern regarding cyclodestructive surgery for glaucoma is a long-term stable IOP. In this experiment, the corneas of the rabbits were damaged by the long-term effects of high IOP, which affected IOP measurements and thus limited the follow-up time after RFA. Therefore, in subsequent clinical trials, the longer-term efficacy and safety of this surgery in patients should be evaluated. Second, various degrees and types of glaucoma are treated in clinical practice, and a single animal model cannot cover its complex manifestations. The RFA parameters used in this animal experiment can be used as a reference for clinical trials, which require careful grouping and further parameter adjustments. Third, the advantages of RFA as a novel treatment method for glaucoma need to be verified through comparisons with traditional approaches and other new methods, such as TCP and HIFU.

## Conclusion

In summary, the RFA device can cause sustained histological damage to the ciliary processes by rapidly raising the local tissue temperature without damaging adjacent eye tissue, leading to epithelial cell apoptosis, subsequently reducing aqueous humor production, and ultimately lowering IOP. Ciliary body RFA as a treatment for refractory glaucoma appears to be a feasible, effective, and well-tolerated method for lowering IOP.

## Methods

### Ciliary body RFA device

The device is chiefly composed of three parts: puncture electrode, radio frequency instrument, and UBM (Fig. 1). The puncture needle has a diameter of 0.2 mm and a length of 1.5 mm to ensure minimal invasion (Fig. 1B). The outer layer of the needle is wrapped with an insulating sheath so that the ablative effect occurs only in the tissues around the tip of the needle, optimizing the targeting and preventing damage to the sclera and other tissues around the corneoscleral border through the puncture path (Fig. 1B). RFA was performed by electrode puncture into the sclera 1.5 mm outside the limbus of the experimental eye, to a depth of about 1.5 mm. After 3D printing, the voltage regulator, ablation energy output device, display panel, foot switch, and other components were combined into an integrated machine, in which the ablation frequency and radiofrequency output mode could be adjusted in real time, making operation more convenient and ensuring accurate and effective ablation damage (Fig. 1E). A self-designed lens–water bath transformation device (Patent No.: ZL201420207350.0) (Fig. 1A) was used to realize the positioning and facilitate real-time observation of the ciliary body during RFA. The test eye was positioned upward, with a lubricating carbomer solution applied to the cornea. The lens–water bath transformation device was used to cover the surface of the eye for UBM examination (Fig. 1D). A puncture electrode was inserted, and the puncture direction and depth were adjusted under UBM guidance, while ciliary body ablation was monitored in real time (Fig. 1C). The device used in this study was specially designed for the anatomy of the rabbit eye. The frequency, power, and duration of each ablation are adjustable.

### Animals

Male New Zealand White rabbits, aged 4 to 6 months, were provided by Beijing Long’an Experimental Animal Breeding Center. The rabbits weighed 2.0 kg to 2.5 kg and were free of eye diseases. The feed and drinking water were sterilized and abundantly provided. Natural lighting was provided, and the indoor temperature was controlled at approximately 25 °C. The rabbits were anesthetized using a mixture of O_2_ and isoflurane (1–4%) via inhalation. Topical anesthesia in the form of oxybuprocaine hydrochloride eye drops (Santen Pharmaceutical Co., Ltd, Japan) was administered to the conjunctival sac, along with an intramuscular injection of xylazine hydrochloride (HUAMU, Jilin, China) at a dose of 0.15 mL/kg.

### Ethics declarations

All procedures were performed in accordance to the relevant guidelines and regulations, and in accordance with the ARRIVE guidelines (Animal Research: Reporting of In Vivo Experiments) to ensure rigorous and transparent reporting (https://www.nc3rs.org.uk/arrive-guidelines). The experimental protocols were approved by the Ethics Committee of Chinese PLA General Hospital (Approval No. [KY2021-022]). Efforts were made to minimize animal suffering and reduce the number of animals used.

### Establishment of the glaucoma model

Previous studies have shown that intracameral injection of silicone oil elevates IOP by blocking the pupil and aqueous humor drainage in animal breeds such as mice and rhesus macaques^27–30^. We hypothesized that direct injection of silicone oil into the anterior chamber could also induce high IOP in rabbits. After general and topical anesthesia, an eyelid speculum was used to open the rabbit’s eyelid and prevent interference by the third eyelid. A 26G needle was used to puncture the sclera 1.5 mm posterior to the limbus of the supratemporal side to reach the anterior chamber without injuring the lens or iris, ensuring that the longer puncture tunnel would self-seal under the IOP to minimize the spillage of silicone oil from the puncture hole (Fig. 2A, B). Silicone oil was injected slowly into the anterior chamber through a silicone oil injector attached to the 26G needle until the oil droplet expanded to cover about 80% of the anterior chamber (Fig. 2C). Meanwhile, at the limbus of the opposite cornea, 0.2 mL of aqueous humor was withdrawn, using a 31G needle. The puncture hole was clamped with the forcep for several seconds to reduce the spillage of silicone oil and facilitate closure of the puncture tunnel when the 26G needle was withdrawn. Fundus photography was performed to observe the changes in the optic disc, and live AS-OCT was performed to observe the state of the anterior chamber angle before and 1 week after silicone oil injection (Fig. 2D–I). The IOP was assessed; IOP > 22 mmHg was used as the standard for a successfully established glaucoma model^26^.

### Grouping of experimental animals

Three groups were established. Model group: glaucoma modeling group. Sham group: glaucoma modeling + sham operation group (the radiofrequency ablation electrode was inserted into the ciliary body under the guidance of UBM, but the electrode did not discharge). RFA group: glaucoma modeling + RFA operation group. Each group consisted of 10 rabbits. The right eye of each rabbit was selected as the treated eye, while the left eye, with normal visual function, was used as the control. The parameters of the RFA group included an output power of 1.6–3 W and a duration of 2 s. Each eye had 14 points treated with ablation. An infrared thermometer was used to monitor the temperature of the electrodes during ablation, and the peak temperature at each ablation site was recorded. The lens–water bath transformation system was used to observe the ablation area via UBM.

### IOP measurement

IOP was measured after the rabbits were anesthetized via inhalation of a mixture of oxygen and isoflurane (1–4%) and administration of 0.4% oxybuprocaine hydrochloride eye drops to the ocular surface. Preoperative IOP was measured using a Schiotz tonometer (Schiotz tonometer, Riester, Germany) and a rebound tonometer (iCare, Tiolat, Finland). Because there was no significant difference in the measured IOP values between the Schiotz tonometer and the rebound tonometer, we used the Schiotz tonometer, which achieved more stable IOP values, for all postoperative measurements. The researcher measuring IOP was blinded to the group of rabbits being tested. If the standard deviation (displayed by the tonometer) of a series exceeded 5%, the measurements were repeated until the standard deviation of each series was less than or equal to 5%.

### Histopathology

#### HE

The pathological changes in the ablated area of the rabbit eye were analyzed by the standard HE staining method. Rabbit eye specimens were obtained and immersed in eye fixative (Servicebio, Wuhan, China) at room temperature overnight. The tissue was dehydrated, embedded in paraffin, and cut into 5 μm thick sections. The sections were stained with hematoxylin–eosin dye according to the manufacturer’s instructions.

#### SEM

The rabbit eyes were sectioned coronally into two parts (anterior segment and posterior segment). The anterior segment was further sectioned in the coronal plane around the ciliary processes. The lens and iris were then detached and removed. Tissue samples were immersed in electron microscope fixative (Baiqiandu Biotech, B1102) at room temperature for 2 h and then transferred to a 4 °C storage environment. Fixed samples were washed for 15 min with 0.1 M phosphate-buffered saline (PBS, pH 7.4) three times, and then 1% osmium tetroxide (Ted Pella Inc.) was used to fix the samples at room temperature in the dark for 2 h. After washing with PBS three times, the samples were dehydrated with a graded series of alcohol before drying. Samples were then introduced onto conductive carbon film double-sided tape and placed on the ion sputtering instrument sample tray for approximately 30 s of gold spraying before examination by SEM (SU8100, Hitachi, Japan).

#### TEM

Target tissue samples were trimmed to approximately 1 × 1 × 1 mm, quickly immersed in electron microscope fixative (Baiqiandu Biotech, B0012) at 4 °C, and fixed for 2–4 h. The samples were rinsed with PBS (pH 7.4) for 15 min three times and then fixed with 1% osmium tetroxide (Ted Pella Inc.) at room temperature for 2 h. After several PBS rinses, the samples were dehydrated in alcohol at graded concentrations, permeated with 812 embedding agent (SPI, 90,529–77-4) overnight, and polymerized at 60 °C for 48 h. Ultra-thin Sects. (70 nm) were subsequently obtained using an ultramicrotome (UC7, Leica, Germany), stained with uranyl acetate and lead citrate solutions, and examined by TEM (HT7800, Hitachi, Japan).

#### TUNEL assay

To evaluate cell apoptosis, paraffin Sects. (5 µm) of ciliary processes were subjected to routine dewaxing and hydration treatment, followed by analysis using the TUNEL BrightRed detection kit (Vazyme, Nanjing, China). The positive control was pretreated with DNase I. After the tissue sections were incubated with 50 µL DAPI (Invitrogen, Carlsbad, CA) at room temperature for 5 min, they were imaged using a confocal microscope (Nikon Ti A1, Tokyo, Japan), and the cells were counted manually. The nuclei of TUNEL-positive apoptotic cells were stained red, while the nuclei of TUNEL-negative cells were stained blue.

### Statistical analysis

Data analysis was performed in GraphPad Prism 9 (GraphPad Prism Software Inc.). Testing for normality was performed using Shapironed blue.c. If the data were normally distributed and homoscedastic, one-way ANOVA was used for statistical analysis. If not, the Kruskal–Wallis test was used. Statistical analyses of IOP were made using the Kruskal–Wallis test. P values less than 0.05 were considered statistically significant.

## Data Availability

Data is provided within the manuscript. Raw data in this study is available from the corresponding authors upon reasonable request.

## Declarations

### Prior publication

This submission has not been published anywhere previously, and it is not simultaneously being considered for any other publication.
